# Editorial: Cancer care in areas of conflict

**DOI:** 10.3389/fonc.2023.1301552

**Published:** 2023-10-31

**Authors:** Omar Shamieh, Tezer Kutluk, Fouad M. Fouad, Richard Sullivan, Asem Mansour

**Affiliations:** ^1^ Center for Palliative & Cancer Care in Conflict (CPCCC), King Hussein Cancer Centre (KHCC), Amman, Jordan; ^2^ Department of Palliative Care, King Hussein Cancer Centre (KHCC), Amman, Jordan; ^3^ Faculty of Medicine, the University of Jordan, Amman, Jordan; ^4^ Department of Pediatric Oncology, Hacettepe University Faculty of Medicine and Cancer Institute, Ankara, Türkiye; ^5^ Department of Epidemiology & Population Health, the American University of Beirut, Beirut, Lebanon; ^6^ Institute of Cancer Policy & Conflict & Health Research Group, King’s College London, London, United Kingdom; ^7^ Department of Radiology, King Hussein Cancer Centre (KHCC), Amman, Jordan

**Keywords:** cancer, conflict, challenges, prevention, diagnosis, treatment, quality of life, palliative care

The global incidence of cancer is witnessing a substantial surge within low- and middle-income countries (LMIC). Projections for the year 2035 indicate that approximately two-thirds of all cancer cases will emerge in these developing nations, with a notable concentration in the Middle East and North Africa (MENA) (1). The MENA region has faced more frequent and severe conflicts than any other part of the world (2). Already grappling with a complex landscape of acute and chronic conflicts, it faces formidable challenges in providing adequate cancer care services to its population. These challenges encompass the absence of universal healthcare coverage, the scarcity of comprehensive cancer control initiatives, a shortage of healthcare professionals, and limited access to early detection and treatment options (1, 3, 4). Furthermore, the enduring armed conflicts and insecurity that pervade the region have directly contributed to widespread migration and the disintegration of healthcare systems, resulting in a stark deficiency of high resolution, accurate healthcare data (1, 5, 6).

This editorial piece delves into the critical subject of cancer care within regions impacted by conflict, drawing insights from narrative synthesis of a compendium of 17 published articles spanning diverse corners of the globe, particularly the Middle East, North Africa, and Turkey (MENAT) area. These articles form a rich tapestry of experiences, encapsulating challenges, obstacles, needs, inequalities, and recommendations related to cancer prevention, diagnosis, treatment, quality of life, and palliative care. Through the lens of these profound contributions, a more profound comprehension of the intricate dynamics impacting cancer care in conflict-affected populations and geographies emerges. By amalgamating the collective knowledge and expertise shared within these articles, the objective is to nurture evidence-based strategies and interventions that address distinctive requisites and elevate outcomes for individuals grappling with cancer amid the adversities of conflict.

One pivotal study on this subject by Daubman et al. spotlighted individuals with cancer and other severe ailments, elucidating their heightened vulnerability during humanitarian emergencies and crises (HECs). The authors underscored the indispensability of palliative care (PC) as an integral facet of holistic care for these patients, a need that amplifies significantly during HECs. The study advocated for the inclusion of PC within humanitarian response policies and guidance, universal training of humanitarian responders in PC, and integration of PC indicators within HECs research. This study shed light on the sustained feasibility of providing the WHO essential package of PC even when other services falter.

A pioneering contribution by Al-Ibraheem et al. navigated the terrain of cancer diagnosis in conflict-afflicted territories of the Middle East, spotlighting the hurdles and limitations experienced in nations like Iraq, Syria, Yemen, and Sudan. The authors advocated surmounting these challenges and formulating tailored approaches to enrich cancer diagnosis in these contexts. Their call for a collaborative, coordinated endeavour at both national and international levels to bridge the gap in cancer diagnosis services reverberated widely across the literature.

Rihani et al. illuminate the hurdles faced by Jordan in providing high-quality cancer care to displaced children, with a specific focus on its role as a prominent host for refugees. Over the period spanning 2011 to 2022, the King Hussein Cancer Center (KHCC) extended care to 968 pediatric patients from outside of Jordan. Remarkably, a substantial 84% of these treatments were financially supported by the King Hussein Cancer Foundation (KHCF). A collaboration with St. Jude Children’s Research Hospital further enhanced care from 2018-2022, benefiting 51 displaced children. This underscores the role of local-international partnerships in bridging healthcare gaps for displaced populations, emphasizing the need for sustainable, innovative care strategies.

Erashi et al. and Mansour et al. approached this topic from a different vantage point, offering insights into the characteristics of Libyan and Palestinian patients under treatment at Jordan’s King Hussein Cancer Center (KHCC), situated within a middle-income MENA country. Adult patients from both populations predominantly exhibited breast, gastrointestinal, and hematolymphoid cancers, with paediatric patients frequently presenting hematolymphoid and central nervous system malignancies. Intriguingly, the presented cases manifested more advanced stages compared to their Jordanian counterparts. Obeidat et al. further contributed by delineating central nervous system tumor attributes among patients from conflict areas treated at KHCC, indicating the prevalence of gliomas, glioneuronal tumors, and neuronal tumors, with a significant proportion being grade 4 tumors.

In the context of the Syrian humanitarian crisis, Yousef et al. conducted a retrospective comparative study on retinoblastoma cases in Syrian refugees and Jordanian citizens treated at KHCC. The study highlighted disparities in age at diagnosis, treatment initiation lag, and disease stage at presentation, emphasizing the urgency of early referrals for optimal management of refugees with retinoblastoma.

Al-Hussaini et al. scrutinized the impact of the COVID-19 pandemic on patients from conflict zones treated at KHCC, uncovering fluctuations in admissions during lockdowns and their subsequent easing. Guo et al. delved into the unique needs of patients with advanced cancer and their caregivers, accentuating the significance of social support and religious considerations in refugee communities. This emphasized the importance of tailoring care to meet these nuanced requirements.

Boufkhed et al. conducted a multicenter qualitative study to uncover priorities and concerns of children and young people (CYP) with advanced cancer and their families in Jordan and Turkey. The findings underlined crucial aspects like physical pain, psychological well-being, spirituality, and social impacts, providing valuable insights into providing tailored, holistic care.Alarjeh et al. further explored communication preferences among Jordanian CYP, caregivers, and healthcare professionals, showcasing the capacity of children and parents to participate in sensitive conversations about care decisions.

Mahmood et al. analysed the demographic and treatment outcome data of Afghan cancer patients seeking treatment in Pakistan, shedding light on the challenges faced and treatment outcomes achieved, emphasizing the need for improved follow-up mechanisms. Lastly, Sater et al. presented a bibliometric analysis of cancer research within conflict-affected settings in the MENA region, identifying trends and calling for targeted research, enhanced research infrastructure, and collaboration to advance cancer outcomes.

In summary, this Research Topic encapsulates a mosaic of experiences, challenges, and advancements within the realm of cancer care amidst conflict. Through these studies, a deeper understanding emerges, offering a springboard for evidence-based interventions that can navigate the complexities of cancer care in regions beset by conflict.

## Author contributions

OS: Conceptualization, Data curation, Investigation, Methodology, Project administration, Supervision, Validation, Writing – original draft, Writing – review & editing. TK: Supervision, Writing – review & editing. FF: Supervision, Writing – review & editing. RS: Supervision, Writing – review & editing. AM: Conceptualization, Supervision, Writing – original draft, Writing – review & editing.
